# Clinical Transplantation and Tolerance: Are We There Yet?

**Published:** 2014-11-01

**Authors:** R. F. Saidi, S. K. Hejazii Kenari

**Affiliations:** Division of Organ Transplantation, Department of Surgery, Alpert Medical School of Brown University, Providence, RI, USA

**Keywords:** Immunosuppression, Transplantation, Childhood, Tolerance, Kidney disease, Neoplasms, Immune system

## Abstract

Organ transplantation is not only considered as the last resort therapy but also as the treatment of choice for many patients with end-stage organ damage. Recipient-mediated acute or chronic immune response is the main challenge after transplant surgery. Nonspecific suppression of host immune system is currently the only method used to prevent organ rejection. Lifelong immunosuppression will cause significant side effects such as infections, malignancies, chronic kidney disease, hypertension and diabetes. This is more relevant in children who have a longer life expectancy so may receive longer period of immunosuppressive medications. Efforts to minimize or complete withdrawal of immunosuppression would improve the quality of life and long-term outcome of pediatric transplant recipients.

## INTRODUCTION

Organ transplantation is not only considered as the last resort therapy but also as the treatment of choice for many patients with end-stage organ damage. Recipient-mediated acute or chronic immune response is the main challenge after transplant surgery. Nonspecific suppression of host immune system is currently the only method used to prevent organ rejection. Lifelong immunosuppression will cause significant side effects such as infections, malignancies, chronic kidney disease, hypertension, and diabetes [1]. This is more relevant in children who have a longer life expectancy so may receive longer period of immunosuppressive medications. Efforts to minimize or complete withdrawal of immunosuppression would improve the quality of life and long-term outcome of pediatric transplant recipients [2]. The current challenges for continuous immunosuppression therapy are its chronic side effects, cost and non-compliance.

**SIDE EFFECTS OF IMMUNOSUPPRESSION**

There are various studies estimating the consequences of post-transplantation non-specific immunosuppression, either by side effect of immunosuppression or as a complication of a defect in body’s defense mechanism.

The type and dosage of most immunosuppressive agents after transplantation have been changed during past decades [5, 6]. For example while usages of cyclosporine and azathoprine have decreased from 1995 to 2005, usage of mycophenolate and tacrolimus have increased. In 2005, 92% of post-transplant patients received tacrolimus [5, 6].

Immunosuppressive therapy can be divided into induction and maintenance therapy. Table 1 shows some of the medications that more commonly used for post-transplantation immunosuppression along with their most common side effects [3, 7].

**Table 1:** Common side effects of immunosuppressants

	Medication	Side Effects
Medication used for induction therapy	Anti-CD25 receptor antibodies (basiliximab, daclizumab)	Anaphylaxis, allergic reaction
	Anti-CD52 monoclonal antibody (alemtuzumab)	T-cell depletion, which increases the risk of infection, in particular CMV reactivation
	Anti-thymocyte globulin (ATG)	Lymphopenia, Serum sickness, anaphylactic reaction,shock,bronchospasm
	Corticosteroids	Cushinoid appearance, fluid retention, diabetes mellitus, hypertension, growth impairment, hyperlipidemia, osteopenia, impairment in wound healing, Failure to thrive
Medication used for maintenance therapy	Calcineurin inhibitors (CNI)	Nephrotoxicity, neurotoxicity, hypertension, hyperlipidemia and hyperkalemia, diabetes mellitus, increased bone resorption, hirsutism, gingival hyperplasia, hearing impairment and cholestatic syndrome
	Azathioprine	Hepatic nodular hyperplasia, Portal sclerosis, Myelosuppression
	Mycophenolate	GI disturbance, myelosuppression growth retardation

Two groups of medications that are well-known to cause direct side effects but yet are the backbone of all immunosuppression therapies, are calcineurin inhibitors (CNI) and corticosteroids. However, current efforts are minimizing their adverse effects by close drug monitoring and multiple drug combination; we cannot fully inhibit their side effects [7].

Corticosteroids put the patients under the risk of developing a wide range of medical problems from poor wound healing, susceptibility to infections, cardiovascular risk factors such as hypertension (by up regulating of α_1_ receptors), and hyperlipidemia to growth retardation and even changing of appearance (Buffalo hump, moon face, *etc*) [8].

CNI are associated with renal toxicity both in renal and non-renal transplantation [8-10]. Rate of renal dysfunction in pediatric recipients of non-renal transplantation is about 55% [7, 11, 12], 3%–6% of whom may develop end-stage renal disease (ESRD)_[7, 13]. Neurotoxicity, hypertension, hyperlipidemia, diabetes mellitus, and hyperkalemia are other side effects of CNIs [3]. 

Half of heart transplant recipients, 30% of liver recipients [7, 13], and 50% of pediatric kidney recipients also have hyperlipidemia [7]. Neurological disorders affect approximately 20% of liver transplant recipients [7]; risk of developing *de novo* malignancies are 3–5 time higher than normal population [7]. 

The risk of developing post-transplant lymphoproliferative disorder (PTLD), which has close relation with Epstein-Barr virus (EBV) infection, is found to be higher in children comparing to adults as it is more likely that they be EBV-seronegative at the time of transplantation [8]. Most pediatric patients receive transplantation at an age when they have naïve immune system and are seronegative for many viruses including EBV and herpes simplex virus (HSV) [14]. CMV infection, BK virus nephropathy and *Pneumocystis carinii* pneumonia (PCP) are among other complications [15]. 

In general, infection, as the main cause of hospitalization after kidney transplantation, is related to immunosuppression [8, 16]. About 85% of liver transplant patients contract infections (bacterial, viral or fungal), which is the cause of death in 28.4% of them [7]. One report by SPLIT registry indicates that infants have the highest risk of developing infections after liver transplantation among other subgroups [1, 4].

Patient dependency on lifelong non-specific immunosuppression is an unsolved problem after transplantation [17]. Tolerance eliminates the complications of long-term immunosuppression use [8], which is a great challenge for pediatric transplant. It can also improve the patient’s compliance which is the main problem in adolescents with chronic disease [18]. Adolescents are particularly prone to non-compliance with their medical regimen as a result of developing sense of authority and poor judgment at this age [19]. 

**COSTS**

Insurance coverage for long-term immunosuppression medication is a considerable problem. Over 70% of kidney transplant programs report that their patients have serious problems paying their medication costs [20]. More than 68% of all programs report even deaths and graft loss because of cost-related immunosuppression non-adherence. However, these problems are more significant in adults than pediatrics, but even children and their families are potentially at risk of facing these problems [20]. In average, the annual cost of immunosuppression is US$ 10,000–15,000. 

**NON-ADHERENCE**

Daily usage of immunosuppression medications may affect the mental health of patients particularly adolescents and their families. Both of these groups are prone to developing psychiatry problems such as depression, post-traumatic stress disorders (PTSD) and other anxiety disorder [19]. Adolescents have the worst outcome of graft survival mainly as a result of non-compliance [15]. Education about the potential risk of non-adherence is challenging in this group. Less complex medical regimen, medication with less side effects and cosmetic change can potentially be more successful [19]. Achieving the state of tolerance however seems to be the best possible solution to overcome adolescent non-adherence.

**WHAT IS TOLERANCE?**

Tolerance is the Holy Grail of transplantation. The concept of tolerance was first introduced in 1953 when Billingham, *et at*, showed that *in utero* injection of bone-marrow cells to mice resulted in acceptance of skin graft from the same inbred donor while maintaining the ability to reject grafts from other breeds [19, 20]. 

In “true tolerance,” the transplant functions normally for a durable time and the recipient is also immunosuppression-free [18], and there is no detectable immune response to the donor antigens [21]. It is defined as a permanent and specific immunological acceptance of allograft antigens without using immunosuppressants [22]. 

“Operational tolerance” is defined as the absence of rejection with normally functional allograft while the patients is immunosuppression-free but it does not necessarily mean lack of immune response towards the graft but rather the lack of destructive response [22]. In operational tolerance there may be some immune response but it has no significant clinical presentation [21]. Operational tolerance usually results from elective or non-elective immunosuppression withdrawal. 

Prope or near tolerance is a term used when allograft functions normally and has normal histology but patients receive minimal immunosuppression [22]. Tolerance induction, theoretically is a method of modification of host immune system in a manner that it does not reject the organ transplanted, but being otherwise normally functional and competent, can result in long-term survival of both the organ and the patient. 

Definition of tolerance in animal models differs as of clinical one. It is defined as acceptance of a graft without immunosuppression use ability to accept subsequent graft from the same donor while having the ability to reject an organ from a third party [23]. 

**PRECLINICAL EXPERIENCE FOR TOLERANCE INDUCTION**

Tolerance is in fact a process not a sudden event [21]. Interestingly, most cases of reported operational tolerance after kidney transplantation are the result of non-compliance. The usual result of discontinuation is graft rejection; nonetheless, some patients do not reject the organ [21].

In 2007 Koyama, *et al*, also showed that CD8+ memory T cells can prevent mixed chimerism and tolerance induction in monkeys [15, 24]. While an immunosuppressive regimen had induced mixed chimerism when kidney transplantation and bone marrow transplantation had been performed simultaneously, the same regimen had failed to do so when bone marrow was transplanted after kidney transplantation. After finding that a numerous number of memory T cells remain even after that regimen, they added humanized anti-CD8 monoclonal antibody and depleting CD8+ memory T cells and could achieved tolerance in non-human primates [15, 24]. 

In 1995 Kawai, *et al*, developed a non-myeloablative regimen that could produce mixed chimerism and renal transplant tolerance in monkeys [15, 25]. Adams, *et al*, showed that virally induced alloreactive memory response in mice, is a barrier to tolerance. They showed a base threshold of memory T cells is needed to induce rejection, with CD8+ memory T cells having the main role [15, 26]. Studies on animal and human models revealed several mechanisms that have role in tolerance. Strategies of tolerance induction in general can be summarized as below:

Using of hematopoietic stem cells (HSC) for induction of activated T cells apoptosis

T cell activation without sufficient support (other cytokines, co-stimulatory factors, *etc*) leads to T cell anergy or death. Blood transfusion is a well-known use of hematopoietic cells for tolerance induction [21]. Recipients of blood transfusions have improved graft survival [21].

Using of HSC for induction of chimerism

Homer’s Iliad is probably the first literary that describes Chimera. A monster reared by *Amisodorus* with lion front, snake tail and a goat in the middle [27]. Full chimerism is total replacement of recipient bone marrow with donor’s bone marrow. This process has a high morbidity and mortality, which can exceed that of immunosuppression therapy [21]. Mixed chimerism is defined as making host immune system in a manner that it composed of both donor and recipient cells [18]. To achieve this, host bone marrow is largely preserved but partially replaced by that of donor [21]. By preventing the bone marrow rejection, the donor’s hematopoietic cells populate in the host bone marrow and thymus and cause central deletion of alloreactive cells [21]. 

In new strategies donors are administered granulocyte stimulating factor and then stem cells are collected by plasmapheresis and then injected to host peripheral or portal veins days before surgery.

There are also some reports that indicate after stem cell transplantation and development of chimerism, not only physicians were able to discontinue immunosuppression but also the primary genetic defect, which had caused organ failure, was cured [28]. 

Patients with primary immunodeficiency disorders usually are not suitable candidates for solid-organ transplantation as post-transplantation immunosuppression can worsen their immune system condition [29]. These patients can benefit from chimerism approach using HSC [29].

Aplastic anemia can be developed in up to 28% of orthotropic liver recipients [30]. Bone marrow transplantation is the treatment of choice in this situation. In 1991 Kawahara, *et al*, reported a 6.5-year boy who received cadaveric liver transplantation after hepatic failure due to non-A non-B hepatitis. The patient developed aplastic anemia three weeks post-transplantation. After about two years of treatment with immunosuppression, he underwent bone-marrow transplant from his HLA-identical sister; about three weeks later he developed full chimerism and ultimately weaned off all immunosuppression.

Depletion

In this strategy host lymphocyte depletion is the goal. However, the ideal situation is to deplete only graft-activated cells as aggressive lymphocyte depletion is unavoidable [18]. To achieve the best result, depletion should be done before graft reperfusion to minimize the number of inflammatory signal producing cells [18].

Different agents are used for this procedure either in preclinical or clinical trials. CD3-dyphteria immunotoxin and deoxy spergualin either as immunotherapy or combined therapy and Campath-1H are some examples.

Altering the co-stimulatory signal

T cells normally require two signals to become activated. The first signal (antigen-specific signal) is provided through interactions between T cell receptor and MHC molecules on antigen-presenting cells (APCs). The second (co-stimulatory) signal is provided by the interaction between molecules expressed on the membrane T cell. There are several well-known co-stimulatory pathways, altering of which can induce tolerance in animals; CD28-B7 co-stimulatory pathway, in which ligation of CD28 on the surface of T cell to the B7 on the surface of APCs can cause T cell activation, is one example [18]. CTLA4 (CD152)-B7 pathway, which limits T cell activation, and CD154-CD40 pathway are other examples [18].

Thymic transplantation

Thymic transplantation is a way to achieve immunocompetence in children with thymic agenesis (Digeorge syndrome) [21, 31]. T cell maturation in donors transplanted thymus, which presents alloantigen, can result in selective tolerance in rats [21, 32, 33].

It is important to note that while many immunomodulatory strategies can induce tolerance in animals, they fail to achieve the same efficacy in clinic [17]. We cannot always extend preclinical experiments to clinical ones as most preclinical models are in rodents which have almost a naïve immune system comparing to human. However, studies on non-human primates seem to be more applicable to human due to their complex immune system [18]. Different organs have particular tolerance mechanism [34]. It means that we cannot extend the mechanism of tolerance in kidney to that of liver. There is no common transcriptional or blood cell markers between kidney and liver transplant tolerance [34, 35]. In addition, it seems that mechanism of tolerance are age-related [34]. However, some blood gene set have been identified to be common in adult and pediatric liver transplants [14].

**CLINICAL EXPERIENCE IN TOLERANCE INDUCTION**

Tolerance induction has been attempted mostly in adult transplant recipients. Studies conducted in Massachusetts General Hospital (MGH) [36], Stanford University [37], and Northwestern Memorial Hospital [38], show the results of tolerance induction in adults. There are only few studies on tolerance induction in pediatrics.

In a study by Kawai, *et al*, at MGH, five patients (22–46 years old) with ESRD received combined kidney and bone-marrow transplantation from their relatives after receiving a non-myeloablative perioperative regimen. While rejection happened in one of them, the other four patients could be withdrawn from all immunosuppression [36].

Patients with multiple myeloma who develop ESRD are usually not eligible for routine treatment for either of these two diseases [39]. Stem cell transplant which is the main treatment for multiple myeloma rarely indicated for ESRD patients; on the other hand, their malignancy does not let them to be a good candidate for transplantation [39]. Spitzer, *et al*, from MGH reported a trial on seven patients with concomitant multiple myeloma and ESRD who underwent combined HLA-matched kidney and bone-marrow transplantation after being prepared with conditioning regimen. All seven patients developed mixed chimerism. Three patients gained normal or near-normal renal function while being off-immunosuppression, two other patients also had normal renal function but immunosuppression therapy restarted because of chronic GVHD. Two patients died—one because of progressive multiple myeloma and another because of developing therapy-related acute myeloid leukemia.

In another report from Stanford University, 16 adult patients enrolled into a study between 2005 and 2011. All of them received kidney transplant after being conditioned with total lymphoid irradiation and antithymocyte globulin. There was a previous study in 2002 where despite achieving chimerism in three out of four combined kidney and bone-marrow transplant patients, none of them could be withdrawn from immunosuppression. In their latter study, however, eight patients could be withdrawn for 1–3 years, three patients were just withdrawn, and one patient was at the midst of withdrawal. Four patients could not be withdrawn due to return of their underlying disease and failure to achieve chimerism or due to rejection [37]. 

Finally, in a report from Northwestern Memorial Hospital, five out of eight HLA-mismatched kidney and hematopoietic stem cell recipients developed durable chimerism and could be weaned off all immunosuppression one year after transplantation [38].

**TOLERANCE BIOMARKERS**

During past decade, many efforts have been made to identify tolerant-related biomarkers. For example, to detect biomarkers for identifying pediatric patients who have more chance to benefit from immunosuppression minimization or withdrawal (tolerance) Li, *et al*, conducted a study. A set of 13 genes were identified to exist in all of their operational tolerant patients, suggesting a method to more specifically select patients for operational tolerance. In their study, approximately 60% of stable pediatric liver transplant patients had high possibility for tolerance. This in turn suggests that in almost the same percentage of these patients immunosuppression can be minimized or withdrawn [14]

Currently identified or under investigation biomarkers are mostly related to dendritic cell, Treg cells and gene polymorphism [40, 41]. A list of tolerance-related biomarkers that are suggested to have a role in withdrawal and/or rejection are listed in Table 2 [40-43]. 

**Table 2:** Biomarkers which have role in tolerance and/or rejections

Tolerance/Rejection	Increasing (↑frequency)	Decreasing(↓frequency)
Increase chance of tolerance	PD-L1/CD86 ratio pDC/mDC ratio Vδ1/ɤδ T cell ratio HLA-G CD4+CD25+ FOXP3+ IL-10	Vα24+Vβ11 NKT TNF-α
Increase risk of rejection	Cyclex, CD154+ Tc memory cells mDC:pDC ratio IL-23 IL-17	

Mechanism of tolerance seems to be different in various organs. For example, while natural killer-related transcripts have the main role in operational tolerance of the liver, B cell-related ones have importance in operational tolerance after kidney transplants [42].

**WHAT IS THE FUTURE OF TOLERANCE?**

With increasing the number of transplantations and improvement in both surgical technologies and tolerance induction regimens, transplantation is entering a new promising era. Improvements in the efficiency of the donation process, public awareness and live donation, development of standardized operation and post-operation protocols, have made donation and organ transplantation more widespread.

Attempts must be made to achieve several goals, one of which is to recognize tolerance-vulnerable patients. There has been some progress in this regard and some potential biomarkers have been identified [41]. Patients should be selected carefully and those who fulfill the primary criteria must be enrolled. 

It might be interesting to point that in contrast to other solid organs, acute rejection after liver transplantation does not cause any long-term sequela [14]. Patients who develop graft rejection after immunosuppression withdrawal can turn back to normal graft function with immunosuppression reinitiating [16, 44]. Tolerance after liver transplantation is more frequent than other organs [14]. This can be related to its characteristic venous endothelium and vascular architecture and dual blood supply [23, 45]. Some articles report a tolerance rate of about 20% in adult liver transplantations [14, 46-49], and even higher rate in children [14, 42, 50].

Combination of the above-mentioned facts can suggest that liver can be the best organ for study of tolerance induction in pediatrics. In selected cases, immunosuppression withdrawal or minimization after liver transplantation can be achieved more frequently than other organs [14]. 

Since the earliest days of solid organ transplantation, weaning or minimizing immunosuppression has been desirable. Identification of suitable patients who can enroll in this process is still a challenge. Identification of tolerance biomarker is a goal. However, there are still no reliable immunologic parameters to assess the feasibility of immunosuppression weaning. Better understanding of the factors involving in immune system activation and tolerance would allow us to develop tailored immunosuppression treatments in which the medication dosage would be reduced and tolerogenic mechanism promoted. Increasing the frequency of reported successful operational tolerance or tolerance induction works like a driving force for development of new weaning protocols. Further studies must focus on development of gene expression profiling as a mean to detect candidates for immunosuppression weaning. Additionally, safer and less toxic protocols with more success rate must be designed.
